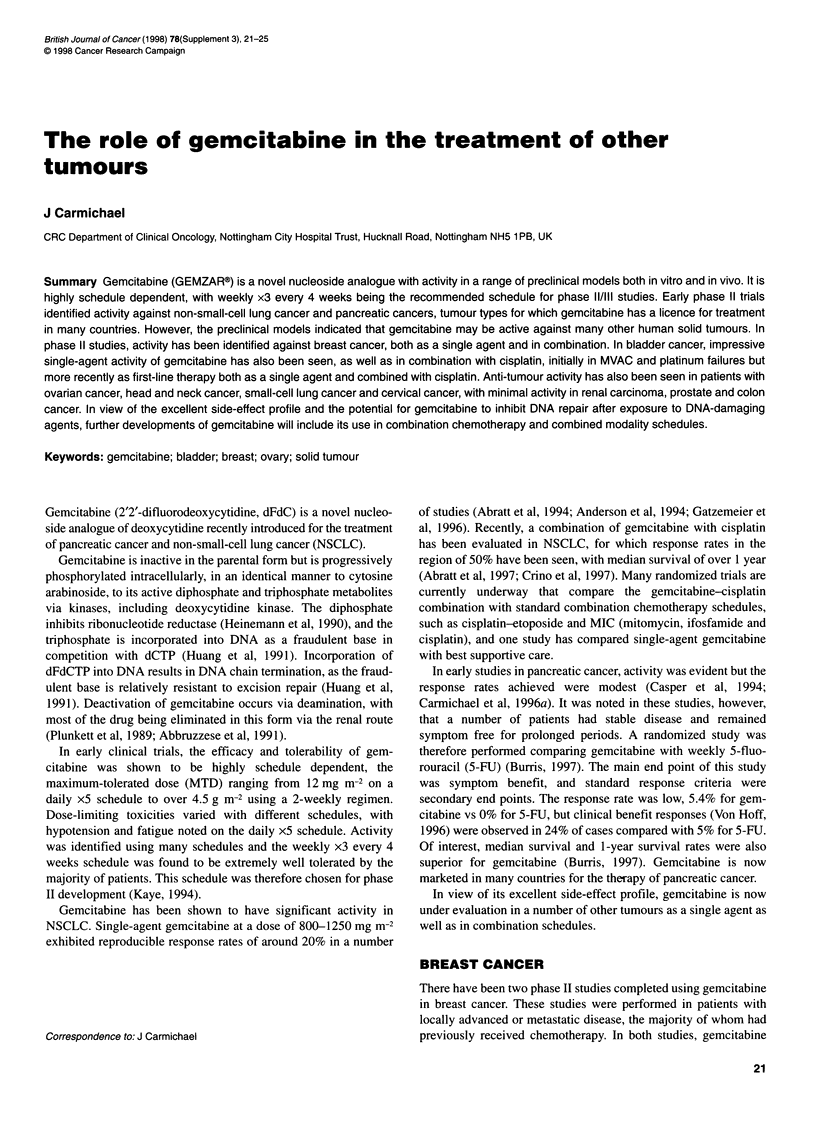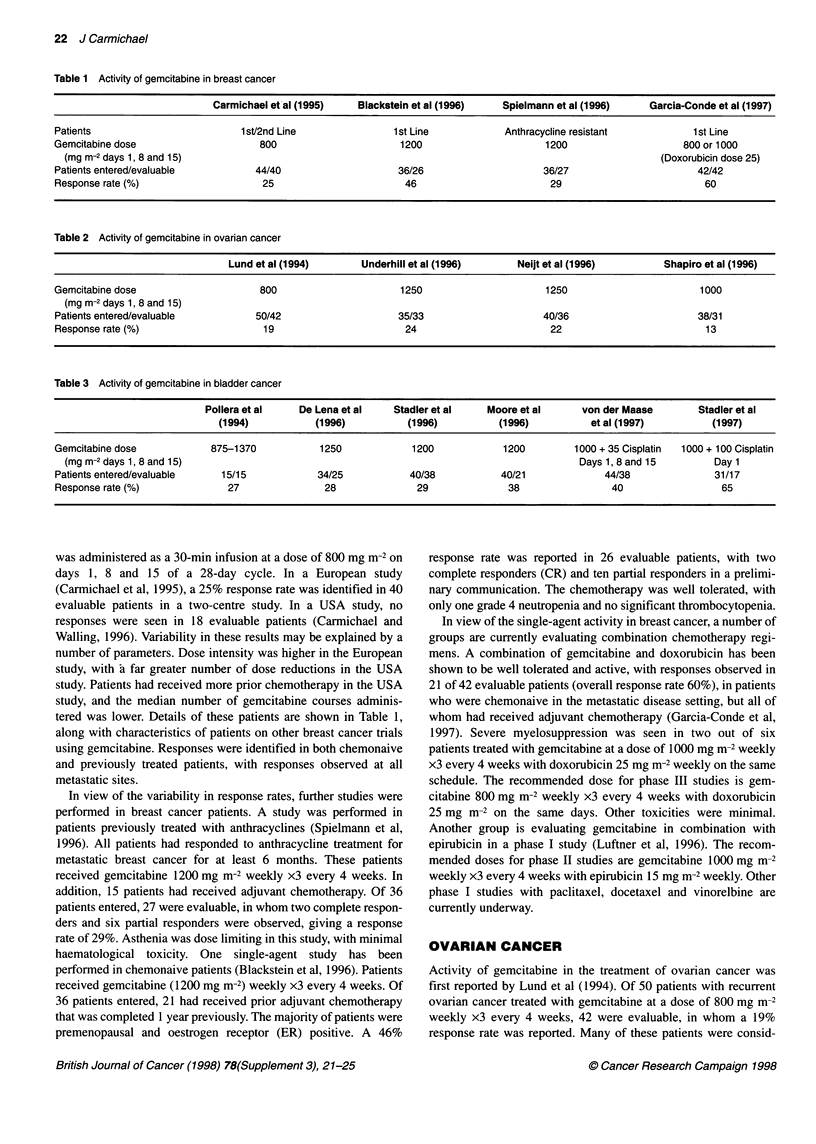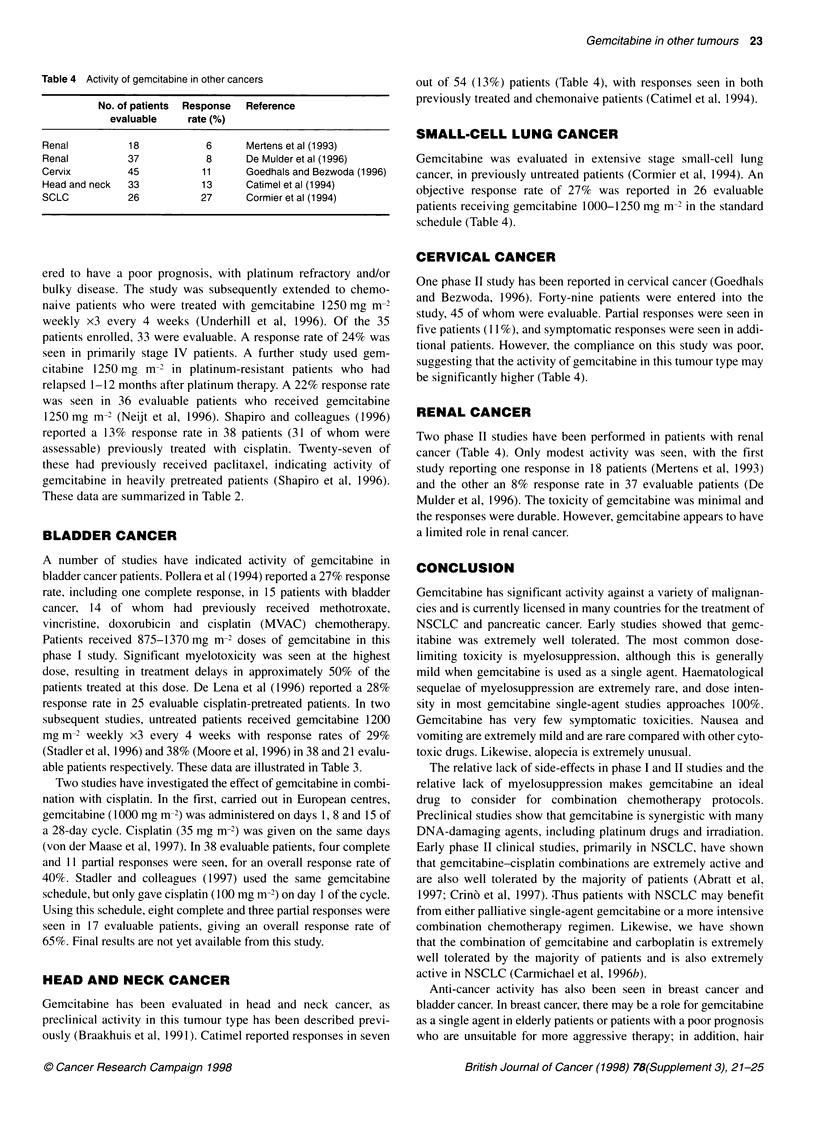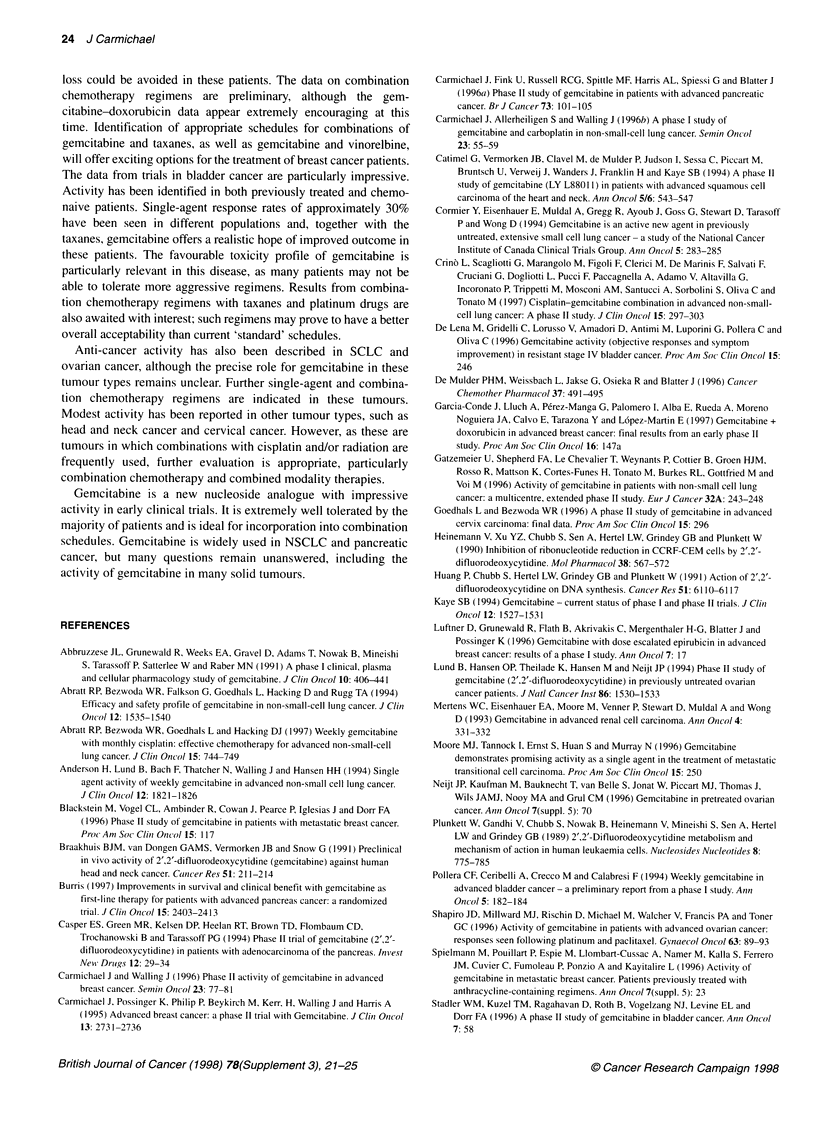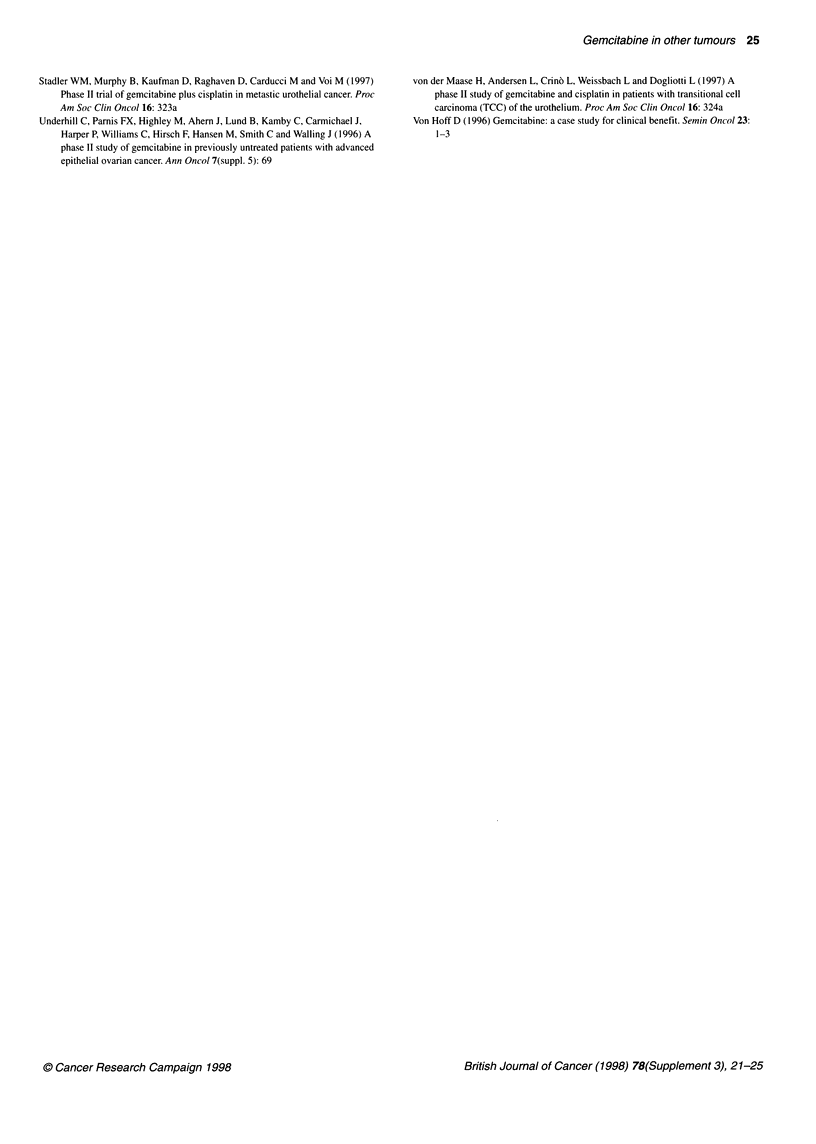# The role of gemcitabine in the treatment of other tumours.

**DOI:** 10.1038/bjc.1998.750

**Published:** 1998

**Authors:** J. Carmichael

**Affiliations:** CRC Department of Clinical Oncology, Nottingham City Hospital, Trust, UK.

## Abstract

Gemcitabine (GEMZAR) is a novel nucleoside analogue with activity in a range of preclinical models both in vitro and in vivo. It is highly schedule dependent, with weekly x3 every 4 weeks being the recommended schedule for phase II/III studies. Early phase II trials identified activity against non-small-cell lung cancer and pancreatic cancers, tumour types for which gemcitabine has a licence for treatment in many countries. However, the preclinical models indicated that gemcitabine may be active against many other human solid tumours. In phase II studies, activity has been identified against breast cancer, both as a single agent and in combination. In bladder cancer, impressive single-agent activity of gemcitabine has also been seen, as well as in combination with cisplatin, initially in MVAC and platinum failures but more recently as first-line therapy both as a single agent and combined with cisplatin. Anti-tumour activity has also been seen in patients with ovarian cancer, head and neck cancer, small-cell lung cancer and cervical cancer, with minimal activity in renal carcinoma, prostate and colon cancer. In view of the excellent side-effect profile and the potential for gemcitabine to inhibit DNA repair after exposure to DNA-damaging agents, further developments of gemcitabine will include its use in combination chemotherapy and combined modality schedules.


British Journal of Cancer (1998) 78(Supplement 3), 21–25
© 1998 Cancer Research Campaign

### The role of gemcitabine in the treatment of other tumours

**J Carmichael**

CRC Department of Clinical Oncology, Nottingham City Hospital Trust, Hucknall Road, Nottingham NH5 1PB, UK

**Summary** Gemcitabine (GEMZAR®) is a novel nucleoside analogue with activity in a range of preclinical models both in vitro and in vivo. It is highly schedule dependent, with weekly ×3 every 4 weeks being the recommended schedule for phase II/III studies. Early phase II trials identified activity against non-small-cell lung cancer and pancreatic cancers, tumour types for which gemcitabine has a licence for treatment in many countries. However, the preclinical models indicated that gemcitabine may be active against many other human solid tumours. In phase II studies, activity has been identified against breast cancer, both as a single agent and in combination. In bladder cancer, impressive single-agent activity of gemcitabine has also been seen, as well as in combination with cisplatin, initially in MVAC and platinum failures but more recently as first-line therapy both as a single agent and combined with cisplatin. Anti-tumour activity has also been seen in patients with ovarian cancer, head and neck cancer, small-cell lung cancer and cervical cancer, with minimal activity in renal carcinoma, prostate and colon cancer. In view of the excellent side-effect profile and the potential for gemcitabine to inhibit DNA repair after exposure to DNA-damaging agents, further developments of gemcitabine will include its use in combination chemotherapy and combined modality schedules.

**Keywords:** gemcitabine; bladder; breast; ovary; solid tumour

Gemcitabine (2′2′-difluorodeoxycytidine, dFdC) is a novel nucleoside analogue of deoxycytidine recently introduced for the treatment of pancreatic cancer and non-small-cell lung cancer (NSCLC).

Gemcitabine is inactive in the parental form but is progressively phosphorylated intracellularly, in an identical manner to cytosine arabinoside, to its active diphosphate and triphosphate metabolites via kinases, including deoxycytidine kinase. The diphosphate inhibits ribonucleotide reductase (Heinemann et al, 1990), and the triphosphate is incorporated into DNA as a fraudulent base in competition with dCTP (Huang et al, 1991). Incorporation of dFdCTP into DNA results in DNA chain termination, as the fraudulent base is relatively resistant to excision repair (Huang et al, 1991). Deactivation of gemcitabine occurs via deamination, with most of the drug being eliminated in this form via the renal route (Plunkett et al, 1989; Abbruzzese et al, 1991).

In early clinical trials, the efficacy and tolerability of gemcitabine was shown to be highly schedule dependent, the maximum-tolerated dose (MTD) ranging from 12 mg $m^{-2}$ on a daily ×5 schedule to over 4.5 g $m^{-2}$ using a 2-weekly regimen. Dose-limiting toxicities varied with different schedules, with hypotension and fatigue noted on the daily ×5 schedule. Activity was identified using many schedules and the weekly ×3 every 4 weeks schedule was found to be extremely well tolerated by the majority of patients. This schedule was therefore chosen for phase II development (Kaye, 1994).

Gemcitabine has been shown to have significant activity in NSCLC. Single-agent gemcitabine at a dose of 800–1250 mg $m^{-2}$ exhibited reproducible response rates of around 20% in a number of studies (Abratt et al, 1994; Anderson et al, 1994; Gatzemeier et al, 1996). Recently, a combination of gemcitabine with cisplatin has been evaluated in NSCLC, for which response rates in the region of 50% have been seen, with median survival of over 1 year (Abratt et al, 1997; Crino et al, 1997). Many randomized trials are currently underway that compare the gemcitabine–cisplatin combination with standard combination chemotherapy schedules, such as cisplatin–etoposide and MIC (mitomycin, ifosfamide and cisplatin), and one study has compared single-agent gemcitabine with best supportive care.

In early studies in pancreatic cancer, activity was evident but the response rates achieved were modest (Casper et al, 1994; Carmichael et al, 1996*a*). It was noted in these studies, however, that a number of patients had stable disease and remained symptom free for prolonged periods. A randomized study was therefore performed comparing gemcitabine with weekly 5-fluorouracil (5-FU) (Burris, 1997). The main end point of this study was symptom benefit, and standard response criteria were secondary end points. The response rate was low, 5.4% for gemcitabine vs 0% for 5-FU, but clinical benefit responses (Von Hoff, 1996) were observed in 24% of cases compared with 5% for 5-FU. Of interest, median survival and 1-year survival rates were also superior for gemcitabine (Burris, 1997). Gemcitabine is now marketed in many countries for the therapy of pancreatic cancer.

In view of its excellent side-effect profile, gemcitabine is now under evaluation in a number of other tumours as a single agent as well as in combination schedules.

### BREAST CANCER

There have been two phase II studies completed using gemcitabine in breast cancer. These studies were performed in patients with locally advanced or metastatic disease, the majority of whom had previously received chemotherapy. In both studies, gemcitabine

*Correspondence to:* J Carmichael

**Table 1** Activity of gemcitabine in breast cancer

| | Carmichael et al (1995) | Blackstein et al (1996) | Spielmann et al (1996) | Garcia-Conde et al (1997) |
|---|---|---|---|---|
| Patients | 1st/2nd Line | 1st Line | Anthracycline resistant | 1st Line |
| Gemcitabine dose (mg m$^{-2}$ days 1, 8 and 15) | 800 | 1200 | 1200 | 800 or 1000 (Doxorubicin dose 25) |
| Patients entered/evaluable | 44/40 | 36/26 | 36/27 | 42/42 |
| Response rate (%) | 25 | 46 | 29 | 60 |

**Table 2** Activity of gemcitabine in ovarian cancer

| | Lund et al (1994) | Underhill et al (1996) | Neijt et al (1996) | Shapiro et al (1996) |
|---|---|---|---|---|
| Gemcitabine dose (mg m$^{-2}$ days 1, 8 and 15) | 800 | 1250 | 1250 | 1000 |
| Patients entered/evaluable | 50/42 | 35/33 | 40/36 | 38/31 |
| Response rate (%) | 19 | 24 | 22 | 13 |

**Table 3** Activity of gemcitabine in bladder cancer

| | Pollera et al (1994) | De Lena et al (1996) | Stadler et al (1996) | Moore et al (1996) | von der Maase et al (1997) | Stadler et al (1997) |
|---|---|---|---|---|---|---|
| Gemcitabine dose (mg m$^{-2}$ days 1, 8 and 15) | 875–1370 | 1250 | 1200 | 1200 | 1000 + 35 Cisplatin Days 1, 8 and 15 | 1000 + 100 Cisplatin Day 1 |
| Patients entered/evaluable | 15/15 | 34/25 | 40/38 | 40/21 | 44/38 | 31/17 |
| Response rate (%) | 27 | 28 | 29 | 38 | 40 | 65 |

was administered as a 30-min infusion at a dose of 800 mg m$^{-2}$ on days 1, 8 and 15 of a 28-day cycle. In a European study (Carmichael et al, 1995), a 25% response rate was identified in 40 evaluable patients in a two-centre study. In a USA study, no responses were seen in 18 evaluable patients (Carmichael and Walling, 1996). Variability in these results may be explained by a number of parameters. Dose intensity was higher in the European study, with a far greater number of dose reductions in the USA study. Patients had received more prior chemotherapy in the USA study, and the median number of gemcitabine courses administered was lower. Details of these patients are shown in Table 1, along with characteristics of patients on other breast cancer trials using gemcitabine. Responses were identified in both chemonaive and previously treated patients, with responses observed at all metastatic sites.

In view of the variability in response rates, further studies were performed in breast cancer patients. A study was performed in patients previously treated with anthracyclines (Spielmann et al, 1996). All patients had responded to anthracycline treatment for metastatic breast cancer for at least 6 months. These patients received gemcitabine 1200 mg m$^{-2}$ weekly ×3 every 4 weeks. In addition, 15 patients had received adjuvant chemotherapy. Of 36 patients entered, 27 were evaluable, in whom two complete responders and six partial responders were observed, giving a response rate of 29%. Asthenia was dose limiting in this study, with minimal haematological toxicity. One single-agent study has been performed in chemonaive patients (Blackstein et al, 1996). Patients received gemcitabine (1200 mg m$^{-2}$) weekly ×3 every 4 weeks. Of 36 patients entered, 21 had received prior adjuvant chemotherapy that was completed 1 year previously. The majority of patients were premenopausal and oestrogen receptor (ER) positive. A 46% response rate was reported in 26 evaluable patients, with two complete responders (CR) and ten partial responders in a preliminary communication. The chemotherapy was well tolerated, with only one grade 4 neutropenia and no significant thrombocytopenia.

In view of the single-agent activity in breast cancer, a number of groups are currently evaluating combination chemotherapy regimens. A combination of gemcitabine and doxorubicin has been shown to be well tolerated and active, with responses observed in 21 of 42 evaluable patients (overall response rate 60%), in patients who were chemonaive in the metastatic disease setting, but all of whom had received adjuvant chemotherapy (Garcia-Conde et al, 1997). Severe myelosuppression was seen in two out of six patients treated with gemcitabine at a dose of 1000 mg m$^{-2}$ weekly ×3 every 4 weeks with doxorubicin 25 mg m$^{-2}$ weekly on the same schedule. The recommended dose for phase III studies is gemcitabine 800 mg m$^{-2}$ weekly ×3 every 4 weeks with doxorubicin 25 mg m$^{-2}$ on the same days. Other toxicities were minimal. Another group is evaluating gemcitabine in combination with epirubicin in a phase I study (Luftner et al, 1996). The recommended doses for phase II studies are gemcitabine 1000 mg m$^{-2}$ weekly ×3 every 4 weeks with epirubicin 15 mg m$^{-2}$ weekly. Other phase I studies with paclitaxel, docetaxel and vinorelbine are currently underway.

### OVARIAN CANCER

Activity of gemcitabine in the treatment of ovarian cancer was first reported by Lund et al (1994). Of 50 patients with recurrent ovarian cancer treated with gemcitabine at a dose of 800 mg m$^{-2}$ weekly ×3 every 4 weeks, 42 were evaluable, in whom a 19% response rate was reported. Many of these patients were consid-

© Cancer Research Campaign 1998

Table 4 Activity of gemcitabine in other cancers

| | No. of patients evaluable | Response rate (%) | Reference |
|---|---|---|---|
| Renal | 18 | 6 | Mertens et al (1993) |
| Renal | 37 | 8 | De Mulder et al (1996) |
| Cervix | 45 | 11 | Goedhals and Bezwoda (1996) |
| Head and neck | 33 | 13 | Catimel et al (1994) |
| SCLC | 26 | 27 | Cormier et al (1994) |

ered to have a poor prognosis, with platinum refractory and/or bulky disease. The study was subsequently extended to chemonaive patients who were treated with gemcitabine 1250 mg $m^{-2}$ weekly ×3 every 4 weeks (Underhill et al, 1996). Of the 35 patients enrolled, 33 were evaluable. A response rate of 24% was seen in primarily stage IV patients. A further study used gemcitabine 1250 mg $m^{-2}$ in platinum-resistant patients who had relapsed 1–12 months after platinum therapy. A 22% response rate was seen in 36 evaluable patients who received gemcitabine 1250 mg $m^{-2}$ (Neijt et al, 1996). Shapiro and colleagues (1996) reported a 13% response rate in 38 patients (31 of whom were assessable) previously treated with cisplatin. Twenty-seven of these had previously received paclitaxel, indicating activity of gemcitabine in heavily pretreated patients (Shapiro et al, 1996). These data are summarized in Table 2.

### BLADDER CANCER

A number of studies have indicated activity of gemcitabine in bladder cancer patients. Pollera et al (1994) reported a 27% response rate, including one complete response, in 15 patients with bladder cancer, 14 of whom had previously received methotroxate, vincristine, doxorubicin and cisplatin (MVAC) chemotherapy. Patients received 875–1370 mg $m^{-2}$ doses of gemcitabine in this phase I study. Significant myelotoxicity was seen at the highest dose, resulting in treatment delays in approximately 50% of the patients treated at this dose. De Lena et al (1996) reported a 28% response rate in 25 evaluable cisplatin-pretreated patients. In two subsequent studies, untreated patients received gemcitabine 1200 mg $m^{-2}$ weekly ×3 every 4 weeks with response rates of 29% (Stadler et al, 1996) and 38% (Moore et al, 1996) in 38 and 21 evaluable patients respectively. These data are illustrated in Table 3.

Two studies have investigated the effect of gemcitabine in combination with cisplatin. In the first, carried out in European centres, gemcitabine (1000 mg $m^{-2}$) was administered on days 1, 8 and 15 of a 28-day cycle. Cisplatin (35 mg $m^{-2}$) was given on the same days (von der Maase et al, 1997). In 38 evaluable patients, four complete and 11 partial responses were seen, for an overall response rate of 40%. Stadler and colleagues (1997) used the same gemcitabine schedule, but only gave cisplatin (100 mg $m^{-2}$) on day 1 of the cycle. Using this schedule, eight complete and three partial responses were seen in 17 evaluable patients, giving an overall response rate of 65%. Final results are not yet available from this study.

### HEAD AND NECK CANCER

Gemcitabine has been evaluated in head and neck cancer, as preclinical activity in this tumour type has been described previously (Braakhuis et al, 1991). Catimel reported responses in seven out of 54 (13%) patients (Table 4), with responses seen in both previously treated and chemonaive patients (Catimel et al, 1994).

### SMALL-CELL LUNG CANCER

Gemcitabine was evaluated in extensive stage small-cell lung cancer, in previously untreated patients (Cormier et al, 1994). An objective response rate of 27% was reported in 26 evaluable patients receiving gemcitabine 1000–1250 mg $m^{-2}$ in the standard schedule (Table 4).

### CERVICAL CANCER

One phase II study has been reported in cervical cancer (Goedhals and Bezwoda, 1996). Forty-nine patients were entered into the study, 45 of whom were evaluable. Partial responses were seen in five patients (11%), and symptomatic responses were seen in additional patients. However, the compliance on this study was poor, suggesting that the activity of gemcitabine in this tumour type may be significantly higher (Table 4).

### RENAL CANCER

Two phase II studies have been performed in patients with renal cancer (Table 4). Only modest activity was seen, with the first study reporting one response in 18 patients (Mertens et al, 1993) and the other an 8% response rate in 37 evaluable patients (De Mulder et al, 1996). The toxicity of gemcitabine was minimal and the responses were durable. However, gemcitabine appears to have a limited role in renal cancer.

### CONCLUSION

Gemcitabine has significant activity against a variety of malignancies and is currently licensed in many countries for the treatment of NSCLC and pancreatic cancer. Early studies showed that gemcitabine was extremely well tolerated. The most common dose-limiting toxicity is myelosuppression, although this is generally mild when gemcitabine is used as a single agent. Haematological sequelae of myelosuppression are extremely rare, and dose intensity in most gemcitabine single-agent studies approaches 100%. Gemcitabine has very few symptomatic toxicities. Nausea and vomiting are extremely mild and are rare compared with other cytotoxic drugs. Likewise, alopecia is extremely unusual.

The relative lack of side-effects in phase I and II studies and the relative lack of myelosuppression makes gemcitabine an ideal drug to consider for combination chemotherapy protocols. Preclinical studies show that gemcitabine is synergistic with many DNA-damaging agents, including platinum drugs and irradiation. Early phase II clinical studies, primarily in NSCLC, have shown that gemcitabine–cisplatin combinations are extremely active and are also well tolerated by the majority of patients (Abratt et al, 1997; Crinò et al, 1997). Thus patients with NSCLC may benefit from either palliative single-agent gemcitabine or a more intensive combination chemotherapy regimen. Likewise, we have shown that the combination of gemcitabine and carboplatin is extremely well tolerated by the majority of patients and is also extremely active in NSCLC (Carmichael et al, 1996*b*).

Anti-cancer activity has also been seen in breast cancer and bladder cancer. In breast cancer, there may be a role for gemcitabine as a single agent in elderly patients or patients with a poor prognosis who are unsuitable for more aggressive therapy; in addition, hair

© Cancer Research Campaign 1998 

loss could be avoided in these patients. The data on combination chemotherapy regimens are preliminary, although the gemcitabine–doxorubicin data appear extremely encouraging at this time. Identification of appropriate schedules for combinations of gemcitabine and taxanes, as well as gemcitabine and vinorelbine, will offer exciting options for the treatment of breast cancer patients. The data from trials in bladder cancer are particularly impressive. Activity has been identified in both previously treated and chemonaive patients. Single-agent response rates of approximately 30% have been seen in different populations and, together with the taxanes, gemcitabine offers a realistic hope of improved outcome in these patients. The favourable toxicity profile of gemcitabine is particularly relevant in this disease, as many patients may not be able to tolerate more aggressive regimens. Results from combination chemotherapy regimens with taxanes and platinum drugs are also awaited with interest; such regimens may prove to have a better overall acceptability than current 'standard' schedules.

Anti-cancer activity has also been described in SCLC and ovarian cancer, although the precise role for gemcitabine in these tumour types remains unclear. Further single-agent and combination chemotherapy regimens are indicated in these tumours. Modest activity has been reported in other tumour types, such as head and neck cancer and cervical cancer. However, as these are tumours in which combinations with cisplatin and/or radiation are frequently used, further evaluation is appropriate, particularly combination chemotherapy and combined modality therapies.

Gemcitabine is a new nucleoside analogue with impressive activity in early clinical trials. It is extremely well tolerated by the majority of patients and is ideal for incorporation into combination schedules. Gemcitabine is widely used in NSCLC and pancreatic cancer, but many questions remain unanswered, including the activity of gemcitabine in many solid tumours.

### REFERENCES

Abbruzzese JL, Grunewald R, Weeks EA, Gravel D, Adams T, Nowak B, Mineishi S, Tarassoff P, Satterlee W and Raber MN (1991) A phase I clinical, plasma and cellular pharmacology study of gemcitabine. *J Clin Oncol* **10**: 406–441

Abratt RP, Bezwoda WR, Falkson G, Goedhals L, Hacking D and Rugg TA (1994) Efficacy and safety profile of gemcitabine in non-small-cell lung cancer. *J Clin Oncol* **12**: 1535–1540

Abratt RP, Bezwoda WR, Goedhals L and Hacking DJ (1997) Weekly gemcitabine with monthly cisplatin: effective chemotherapy for advanced non-small-cell lung cancer. *J Clin Oncol* **15**: 744–749

Anderson H, Lund B, Bach F, Thatcher N, Walling J and Hansen HH (1994) Single agent activity of weekly gemcitabine in advanced non-small cell lung cancer. *J Clin Oncol* **12**: 1821–1826

Blackstein M, Vogel CL, Ambinder R, Cowan J, Pearce P, Iglesias J and Dorr FA (1996) Phase II study of gemcitabine in patients with metastatic breast cancer. *Proc Am Soc Clin Oncol* **15**: 117

Braakhuis BJM, van Dongen GAMS, Vermorken JB and Snow G (1991) Preclinical in vivo activity of 2′,2′-difluorodeoxycytidine (gemcitabine) against human head and neck cancer. *Cancer Res* **51**: 211–214

Burris (1997) Improvements in survival and clinical benefit with gemcitabine as first-line therapy for patients with advanced pancreas cancer: a randomized trial. *J Clin Oncol* **15**: 2403–2413

Casper ES, Green MR, Kelsen DP, Heelan RT, Brown TD, Flombaum CD, Trochanowski B and Tarassoff PG (1994) Phase II trial of gemcitabine (2′,2′-difluorodeoxycytidine) in patients with adenocarcinoma of the pancreas. *Invest New Drugs* **12**: 29–34

Carmichael J and Walling J (1996) Phase II activity of gemcitabine in advanced breast cancer. *Semin Oncol* **23**: 77–81

Carmichael J, Possinger K, Philip P, Beykirch M, Kerr, H, Walling J and Harris A (1995) Advanced breast cancer: a phase II trial with Gemcitabine. *J Clin Oncol* **13**: 2731–2736

Carmichael J, Fink U, Russell RCG, Spittle MF, Harris AL, Spiessi G and Blatter J (1996*a*) Phase II study of gemcitabine in patients with advanced pancreatic cancer. *Br J Cancer* **73**: 101–105

Carmichael J, Allerheiligen S and Walling J (1996*b*) A phase I study of gemcitabine and carboplatin in non-small-cell lung cancer. *Semin Oncol* **23**: 55–59

Catimel G, Vermorken JB, Clavel M, de Mulder P, Judson I, Sessa C, Piccart M, Bruntsch U, Verweij J, Wanders J, Franklin H and Kaye SB (1994) A phase II study of gemcitabine (LY L88011) in patients with advanced squamous cell carcinoma of the heart and neck. *Ann Oncol* **5/6**: 543–547

Cormier Y, Eisenhauer E, Muldal A, Gregg R, Ayoub J, Goss G, Stewart D, Tarasoff P and Wong D (1994) Gemcitabine is an active new agent in previously untreated, extensive small cell lung cancer – a study of the National Cancer Institute of Canada Clinical Trials Group. *Ann Oncol* **5**: 283–285

Crinò L, Scagliotti G, Marangolo M, Figoli F, Clerici M, De Marinis F, Salvati F, Cruciani G, Dogliotti L, Pucci F, Paccagnella A, Adamo V, Altavilla G, Incoronato P, Trippetti M, Mosconi AM, Santucci A, Sorbolini S, Oliva C and Tonato M (1997) Cisplatin–gemcitabine combination in advanced non-small-cell lung cancer: A phase II study. *J Clin Oncol* **15**: 297–303

De Lena M, Gridelli C, Lorusso V, Amadori D, Antimi M, Luporini G, Pollera C and Oliva C (1996) Gemcitabine activity (objective responses and symptom improvement) in resistant stage IV bladder cancer. *Proc Am Soc Clin Oncol* **15**: 246

De Mulder PHM, Weissbach L, Jakse G, Osieka R and Blatter J (1996) *Cancer Chemother Pharmacol* **37**: 491–495

Garcia-Conde J, Lluch A, Pérez-Manga G, Palomero I, Alba E, Rueda A, Moreno Noguiera JA, Calvo E, Tarazona Y and López-Martin E (1997) Gemcitabine + doxorubicin in advanced breast cancer: final results from an early phase II study. *Proc Am Soc Clin Oncol* **16**: 147a

Gatzemeier U, Shepherd FA, Le Chevalier T, Weynants P, Cottier B, Groen HJM, Rosso R, Mattson K, Cortes-Funes H, Tonato M, Burkes RL, Gottfried M and Voi M (1996) Activity of gemcitabine in patients with non-small cell lung cancer: a multicentre, extended phase II study. *Eur J Cancer* **32A**: 243–248

Goedhals L and Bezwoda WR (1996) A phase II study of gemcitabine in advanced cervix carcinoma: final data. *Proc Am Soc Clin Oncol* **15**: 296

Heinemann V, Xu YZ, Chubb S, Sen A, Hertel LW, Grindey GB and Plunkett W (1990) Inhibition of ribonucleotide reduction in CCRF-CEM cells by 2′,2′-difluorodeoxycytidine. *Mol Pharmacol* **38**: 567–572

Huang P, Chubb S, Hertel LW, Grindey GB and Plunkett W (1991) Action of 2′,2′-difluorodeoxycytidine on DNA synthesis. *Cancer Res* **51**: 6110–6117

Kaye SB (1994) Gemcitabine – current status of phase I and phase II trials. *J Clin Oncol* **12**: 1527–1531

Luftner D, Grunewald R, Flath B, Akrivakis C, Mergenthaler H-G, Blatter J and Possinger K (1996) Gemcitabine with dose escalated epirubicin in advanced breast cancer: results of a phase I study. *Ann Oncol* **7**: 17

Lund B, Hansen OP, Theilade K, Hansen M and Neijt JP (1994) Phase II study of gemcitabine (2′,2′-difluorodeoxycytidine) in previously untreated ovarian cancer patients. *J Natl Cancer Inst* **86**: 1530–1533

Mertens WC, Eisenhauer EA, Moore M, Venner P, Stewart D, Muldal A and Wong D (1993) Gemcitabine in advanced renal cell carcinoma. *Ann Oncol* **4**: 331–332

Moore MJ, Tannock I, Ernst S, Huan S and Murray N (1996) Gemcitabine demonstrates promising activity as a single agent in the treatment of metastatic transitional cell carcinoma. *Proc Am Soc Clin Oncol* **15**: 250

Neijt JP, Kaufman M, Bauknecht T, van Belle S, Jonat W, Piccart MJ, Thomas J, Wils JAMJ, Nooy MA and Grul CM (1996) Gemcitabine in pretreated ovarian cancer. *Ann Oncol* **7**(suppl. 5): 70

Plunkett W, Gandhi V, Chubb S, Nowak B, Heinemann V, Mineishi S, Sen A, Hertel LW and Grindey GB (1989) 2′,2′-Difluorodeoxycytidine metabolism and mechanism of action in human leukaemia cells. *Nucleosides Nucleotides* **8**: 775–785

Pollera CF, Ceribelli A, Crecco M and Calabresi F (1994) Weekly gemcitabine in advanced bladder cancer – a preliminary report from a phase I study. *Ann Oncol* **5**: 182–184

Shapiro JD, Millward MJ, Rischin D, Michael M, Walcher V, Francis PA and Toner GC (1996) Activity of gemcitabine in patients with advanced ovarian cancer: responses seen following platinum and paclitaxel. *Gynaecol Oncol* **63**: 89–93

Spielmann M, Pouillart P, Espie M, Llombart-Cussac A, Namer M, Kalla S, Ferrero JM, Cuvier C, Fumoleau P, Ponzio A and Kayitalire L (1996) Activity of gemcitabine in metastatic breast cancer. Patients previously treated with anthracycline-containing regimens. *Ann Oncol* **7**(suppl. 5): 23

Stadler WM, Kuzel TM, Ragahavan D, Roth B, Vogelzang NJ, Levine EL and Dorr FA (1996) A phase II study of gemcitabine in bladder cancer. *Ann Oncol* **7**: 58

© Cancer Research Campaign 1998

Stadler WM, Murphy B, Kaufman D, Raghaven D, Carducci M and Voi M (1997) Phase II trial of gemcitabine plus cisplatin in metastic urothelial cancer. *Proc Am Soc Clin Oncol* **16**: 323a

Underhill C, Parnis FX, Highley M, Ahern J, Lund B, Kamby C, Carmichael J, Harper P, Williams C, Hirsch F, Hansen M, Smith C and Walling J (1996) A phase II study of gemcitabine in previously untreated patients with advanced epithelial ovarian cancer. *Ann Oncol* **7**(suppl. 5): 69

von der Maase H, Andersen L, Crinò L, Weissbach L and Dogliotti L (1997) A phase II study of gemcitabine and cisplatin in patients with transitional cell carcinoma (TCC) of the urothelium. *Proc Am Soc Clin Oncol* **16**: 324a

Von Hoff D (1996) Gemcitabine: a case study for clinical benefit. *Semin Oncol* **23**: 1–3

© Cancer Research Campaign 1998